# Dual Diagnosis of Fragile X Syndrome and DEPDC5-Related Disorder Emphasizes DEPDC5's Role Beyond Familial Epilepsy: A Case Report and Literature Review

**DOI:** 10.1155/crig/4501466

**Published:** 2025-04-02

**Authors:** Rory Edwards, Grace Murphy, Joshua W. Owens, Craig Erickson, Robert Hopkin, Amelle Shillington

**Affiliations:** ^1^University of Cincinnati College of Medicine, Cincinnati, Ohio, USA; ^2^Division of Human Genetics, Cincinnati Children's Hospital Medical Center, Cincinnati, Ohio, USA; ^3^Department of Pediatrics, University of Cincinnati College of Medicine, Cincinnati, Ohio, USA; ^4^Department of Psychiatry and Behavioral Neuroscience, University of Cincinnati College of Medicine, Cincinnati, Ohio, USA

**Keywords:** DEPDC5, dual diagnosis, fragile X syndrome, mTOR inhibitor, neurodevelopmental disorder

## Abstract

Dep domain-containing Protein 5 (DEPDC5), encoded by the gene DEPDC5, regulates the cell cycle by inhibiting the mTORC1 pathway in response to amino acid deficiency. Loss of function DEPDC5 variants are recognized to present as focal familial epilepsy; however, associations with comorbid brain malformations and neurodevelopmental disorders have also been reported. mTOR inhibitors were found to benefit DEPDC5-knockout mice. Fragile X syndrome (FXS) is an X-linked neurodevelopmental disorder caused by loss of function of FMR1, and females are expected to have milder neurodevelopmental presentations than males. The reported individual is a 17-year-old female diagnosed with FXS at 1 year of age, but the severity of her neuropsychiatric symptoms prompted further genetic testing at age 14, revealing a likely pathogenic c.4307_4310del DEPDC5 variant. Following this diagnosis, she was started on the mTOR inhibitor sirolimus without significant clinical response. She has never been diagnosed with epilepsy; however, her DEPDC5 and FXS dual diagnosis was thought explanatory for her presentation. A review of 213 previously reported individuals with DEPDC5-related disorder demonstrated that 15.2% of individuals do not have epilepsy, 24.3% have intellectual disability, and 33.8% have brain malformations. Her lack of response to sirolimus may represent the presence of a critical treatment window for mTOR inhibitors in neurodevelopmental disorders.

## 1. Introduction

DEPDC5 encodes a subunit of the GATOR1 complex, which inhibits the mTORC1 pathway in response to amino acid deficiency to decrease cell growth, proliferation, and migration. Autosomal dominant loss of function DEPDC5 variants are expected to lead to dysregulation and an overall increase in mTOR activity leading to abnormalities in neurogenesis, neural network activity, synaptic transmission, and plasticity [1]. Initial descriptions of pathogenic DEPDC5 variants focused on its link to focal epilepsy, though more recent studies have suggested associations with sleep-related hypermotor epilepsy, infantile spasms, focal cortical dysplasia, developmental encephalopathy, macrocephaly, polymicrogyria, hemimegalencephaly, autism spectrum disorder (ASD), anxiety, and attention deficit hyperactivity disorder (ADHD) [1]. Multiple genetic syndromes within the mTOR pathway cause mTOR upregulation and demonstrate symptom improvement with mTOR inhibitors that complex with FKBP12 to cause allosteric mTORC1 inhibition, and mouse studies suggest that mTOR inhibitors can reduce seizures and increase lifespan in DEPDC5 loss of function models [2].

Fragile X syndrome (FXS) is an X-linked dominant neurodevelopmental disorder caused by loss of function of FMR1. Most cases are due to CGG trinucleotide repeat expansions that cause the 5-prime untranslated region of FMR1 mRNA to hybridize to its complementary DNA repeat region, creating an RNA/DNA duplex that inactivates the gene [3]. FMR1 mediates the gene expression at the synapse, with loss of function leading to impaired synaptic maturation manifesting as global developmental delay (GDD), intellectual disability, ASD, dysmorphic facial features, hypotonia, seizures, hypergonadotropic hypogonadism, and/or ataxia [4]. Males with FXS generally have a higher frequency and severity of symptoms than females, though the full spectrum of FXS can be seen in females [5]. Because females have a normal FMR1 allele on their second X chromosome, they tend to have higher cognitive functioning than their male counterparts. Variability in intellectual disability in females is due to X inactivation of different proportions of cells [6]. One meta-analysis estimated the prevalence of ASD in females with FXS as 14%, compared to 60% in males; however, there is high variability among currently published studies [4].

Here, we present the first case of a 17-year-old female, who had minimal clinical response to a trial of the mTOR inhibitor sirolimus, with a dual diagnosis of FXS and DEPDC5-related disorder. Through a review of the medical literature, we have compiled the largest available DEPDC5 cohort to demonstrate the degree to which DEPDC5-related disorders extend beyond epilepsy to include brain malformations and neuropsychiatric symptoms.

## 2. Case Presentation

This 17-year-old female was the product of an uncomplicated pregnancy and was delivered at term via spontaneous vaginal delivery. She initially fed appropriately and was discharged by the 2nd day of life. By 2 months of age, her family suspected that she may have FXS due to feeding difficulties, GDD, and similarities with her older brother with a known diagnosis of FXS. FXS testing was initially deferred since she was female, but due to persistent GDD at 1 year of age, testing demonstrated a full pathogenic variant of approximately 685 repeats on one X chromosome and less than 34 repeats on her second X chromosome, diagnostic for FXS. A Vineland II Adaptive Behavior Scale assessment at 13 months of age demonstrated a composite score below the 1st percentile. A Child Autism Rating Scale assessment at this time was consistent with a diagnosis of autism.

By age two, she had significant irritability and aggressive behaviors, including head banging requiring a helmet to keep her safe. She also had disrupted sleep, prompting the initiation of melatonin. A clonidine trial led to increased self-injurious behaviors and sedation, prompting a transition to risperidone by 30 months of age.

By age four, she developed hypotonia and joint hypermobility managed with occupational therapy. A trial of sertraline for concerns of anxiety contributing to irritability, and self-injurious behavior showed mild improvement in symptoms. She was intermittently trialed on amphetamine/dextroamphetamine salts and guanfacine for hyperarousal and ADHD symptoms, though these were eventually discontinued due to worsening aggressive behaviors. She had mild improvement while on buspirone.

By 6 years of age, she had ongoing difficulty controlling aggression, outbursts, and irritability, leading to an inpatient psychiatric admission. Risperidone was discontinued due to repetitive eye blinking movements thought to be a symptom of tardive dyskinesia, which resolved within 1 month of discontinuation. She was intermittently trialed on both antiseizure medication (valproic acid, lorazepam, clonazepam, and oxcarbazepine) and neuroleptics (aripiprazole) without clear improvements in her behaviors.

By 10 years of age, she continued to display increased aggression toward herself and others that limited her ability to participate in school. She had ongoing poor sleep, anhedonia, and a decreased appetite. She had an EEG around this time after a 1 min episode of shaking, decreased responsiveness, and perioral cyanosis that resolved spontaneously. The EEG was abnormal for the age with a slow background rhythm but without epileptiform activity. This episode was thought to be nonepileptic.

At age 12, she was trialed on lithium with significant initial improvement in her symptoms. Cognitive testing was repeated at this time, the Adaptive Behavior Assessment System 2nd Edition (ABAS-3) revealed a general adaptive composite score of 40, a conceptual score of 50, a social score of 55, and a practical score of 43. These scores were consistent with a diagnosis of moderate intellectual disability. At age 14, a next generation sequencing panel targeting neurodevelopmental disorders (IDEA Panel, PreventionGenetics) was completed due to concerns that her clinical course was more severe than could be explained by FXS alone. This identified a likely pathogenic variant, DEPDC5(NM_001242896.1 c.4307_4310del, p.Arg1436Profs∗137, het, pat) inherited from her asymptomatic father, which is predicted to cause a frameshift in exon 38 and lead to nonsense mediated decay. Following this new dual diagnosis, a follow-up overnight EEG was recommended; however, it was felt that the patient would not be able to behaviorally tolerate such a study and was thus deferred. An MRI brain was recommended, though it was deferred due to family preference for avoiding anesthesia risk, and the uncertain benefit that the discovery of a cortical brain malformation would impact care as a follow-up EEG would not be possible. Thus, the decision was made to move forward with the targeted therapy for DEPDC5.

Given her lack of response to previous medications, ongoing difficult behaviors, and preliminary evidence suggesting that mTOR inhibitors may improve symptoms of DEPDC5-related disorders, she was trialed on sirolimus shortly after this new dual diagnosis. She was titrated to a goal trough dose of 5.0–15.0 ng/mL, with parents not reporting any significant change in her behavior or negative side effects related to sirolimus.

Roughly 1 year after initiating sirolimus, she was hospitalized for pyelonephritis, hypernatremia, and acute kidney injury (AKI). During this admission, she was found to have nephrogenic diabetes insipidus thought due to side effect of lithium administration. Due to her renal injury, the sirolimus was held and parents subsequently noted no worsening of her behaviors. Since sirolimus was associated with neither benefit nor detriment to her condition, parents elected not to restart the medication trial. She also weaned off lithium without significant changes in her behavior.

Now at age 17, she continues to struggle with aggressive and self-injurious behaviors and has psychiatric diagnoses of disruptive behavior disorder, intermittent explosive disorder, and moderate intellectual disability.

## 3. Discussion

As genetic testing becomes more accessible and informative, identifying individuals with more than one pathogenic variant is becoming more common [7], and the wider spectrum of symptoms associated with monogenic disorders is becoming more apparent. This individual's case demonstrates the importance of recognizing the full phenotypic spectrum of a syndrome and searching for an additional diagnosis when an individual's symptoms fall outside of the suspected symptom severity. Anxiety and mild hypotonia are consistent with a diagnosis of FXS in females, but ASD, ID, and anxiety are documented to occur in both FXS and DEPDC5-related disorders [8]. The overlap of these pathologies is hypothesized to led to a more severe phenotype in this individual and may explain why her neurocognitive and neuropsychiatric symptom presentation was much more severe than expected for females with FXS.

Initial reports linked pathogenic DEPDC5 variants with epilepsy, with rare reports of comorbid neuropsychiatric diagnoses or brain malformations [1]. Given this individual's lack of epilepsy and more severe behavioral symptoms, we reviewed 213 previously reported individuals in the medical literature with DEPDC5-related disorders to better establish the phenotypic spectrum of DEPDC5-related disorders (Table 1) [9–20]. This cohort suggested incomplete penetrance of pathogenic DEPDC5 variants, with 15.2% of individuals reported as asymptomatic. A significant number of individuals had brain abnormalities or neuropsychiatric symptoms, reinforcing DEPDC5-related disorder as having implications beyond epilepsy. Of note, many of the larger publications referenced for this table had cohorts primarily collected from epilepsy centers or as part of screening studies for focal epilepsy, and there has been relatively little emphasis on screening individuals presenting with ID without epilepsy for pathogenic DEPDC5 variants. Thus, we suspect that this cohort may have sampling bias that overestimates rates of epilepsy and underestimates rates of neuropsychiatric features in individuals with DEPDC5-related disorder. This may also contribute to the higher rate of penetrance reported in this cohort, as previous cohorts have suggested that asymptomatic heterozygotes may be more common [17, 18]. More research is needed to determine whether this individual's neuropsychiatric symptoms without epilepsy are a common presentation of pathogenic DEPDC5 variants. Regardless, these data suggest that routine psychiatric and neurobehavioral screening should be performed as a standard of care for individuals with DEPDC5-related disorders.

From ages 14 to 15, this individual received roughly a one-year trial course of sirolimus for newly diagnosed DEPDC5-related disorder. Both tuberous sclerosis complex (TSC) and DEPDC5-related disorders are caused by pathogenic variants in the mTORC1 pathway, which upregulate/dysregulate mTORC1, and frequently present as treatment-refractory epilepsy [1]. mTOR inhibitors have successfully reduced seizure frequency in TSC where more typical antiepileptic drugs have failed, and sirolimus has reduced seizure frequency and extended the lifespan of DEPDC5-knockout mouse models [2]. There is mixed evidence as to the benefit of mTOR inhibitors in neuropsychiatric conditions beyond epilepsy in the mTORopathies, with individual studies showing that mTOR inhibitors have both had no impact on children with ASD and TSC [21, 22], as well as potential improvement of cognitive function in mouse models of TSC [23]. This may be related to a critical window of treatment, as mouse models of mTORopathies demonstrated improved social behaviors with rapamycin therapy initiated at 6 weeks of age (analogous to young adulthood) but not 10 weeks of age (analogous to older adulthood) [24].

The parents and clinicians of this individual did not note any clinically relevant change in behavior or any other side effects when sirolimus was started or after it was discontinued. Although it appeared not to ameliorate symptoms related to her diagnosis of DEPDC5-disorder, sirolimus was able to be safely trialed in this individual. It is possible that sirolimus' lack of efficacy in this individual was due to the timing of administration in late development or duration of dosing of too short a period to impact neurodevelopmental symptoms.

This case report describes symptoms related to the dual diagnosis of FXS and DEPDC5-related disorder and reinforces the importance of seeking an additional genetic diagnosis when a individual is on the most extreme end of a syndrome's spectrum. Sirolimus was safe but lacked clear effectiveness in alleviating behavioral symptoms in this individual, though it may have been successful if trialed at a younger age or for a longer duration. More research is needed to determine the role of mTOR inhibitors in treating DEPDC5-related disorders.

## Figures and Tables

**Table 1:** Comparison of symptom presentation of this individual with a profile of symptoms associated with DEPDC5-related disorder, assembled via the literature review of 213 previously described individuals with DEPDC5 pathogenic variants.

	Reported individual	DEPDC5 cohort (*n* = 213)
Gender	F	99 female (49.0%)
Symptom onset	2 months	Mean 18.2 months (*n* = 45)
Epilepsy	−	178/210 (84.8%)
FH of epilepsy	−	149/198 (75.3%)
FH of SUDEP	−	14/81 (17.3%)
Sleep-related hypermotor epilepsy	−	36/204 (17.7%)
Focal epilepsy	−	103/195 (52.8%)
Frontal	−	43/201 (21.4%)
Temporal	−	32/201 (15.9%)
Infantile spasms	−	16/204 (7.8%)
Rolandic epilepsy	−	5/58 (8.6%)
Generalized epilepsy	−	19/205 (9.3%)
Drug-resistant epilepsy	−	57/98 (58.2%)
Neuropsychiatric features		
Intellectual disability	+	43/177 (24.3%)
Language delay	+	16/176 (9.1%)
ADHD	+	14/173 (8.1%)
ASD	+	12/173 (6.9%)
ODD	−	15/173 (8.7%)
Anxiety	+	5/173 (2.9%)
Other psychiatric disorder	+	10/182 (5.5%)
Brain abnormalities	?	94/142 (33.8%)
Malformations of cortical development	?	36/103 (35.0%)
Focal cortical dysplasia	?	29/103 (28.16%)
Hemimegalencephaly	?	3/103 (2.9%)
Hippocampal sclerosis	?	4/103 (3.9%)

*Note:* −= symptom absent; + = symptom present; ? = no imaging performed. The individual was excluded from a category if the presence or absence of a symptom was not reported, leading to different denominators for each category.

Abbreviations: ADHD, attention-deficit/hyperactivity disorder; ASD, autism spectrum disorder; FH, family history; ODD, oppositional defiant disorder; SUDEP, sudden unexpected death in epilepsy.

## Data Availability

All data used in the creation of this manuscript are available upon request.
